# Genetic diversity of *Helosciadium repens* (Jacq.) W.D.J. Koch (Apiaceae) in Germany, a Crop Wild Relative of celery

**DOI:** 10.1002/ece3.5947

**Published:** 2019-12-17

**Authors:** Tobias Herden, Maria Bönisch, Nikolai Friesen

**Affiliations:** ^1^ Botanical Garden of the Osnabrueck University Osnabrueck Germany; ^2^ Federal Research Center for Cultivated Plants Julius Kühn‐Institute Quedlinburg Germany; ^3^ Department of Pharmaceutical and Natural Sciences Ministry of Health of the Russian Federation I. M. Sechenov First Moscow State Medical University Moscow Russia

**Keywords:** Crop Wild Relatives, genetic diversity, genetic reserves, *Helosciadium repens*, most appropriate wild population, SSR

## Abstract

- *Helosciadium repens* (Jacq.) W.D.J. Koch is threatened by genetic erosion. It is a Crop Wild Relative (CWR) of celery and celeriac and a potentially valuable genetic resource for plant breeding. The objective of this study was the analysis of distribution of genetic diversity with a set of selected populations in Germany. The results of the genetic analysis and data obtained during the site visits were used to identify a subset which was chosen to best represent the genetic diversity of *H. repens* in Germany. The chance of long‐term conservation by securing the identified populations in genetic reserves is distinctly possible.
- Seven hundred and fifteen individuals from 27 sites were assessed using six simple sequence repeat markers. Discriminant analysis of principal components was used to identify six clusters of genetically similar individuals. The complementary compositional genetic differentiation Δ*j* was calculated to designate a subset of populations chosen to best represent the overall genetic diversity. Entry 18R (Δ_18R_ = 0.2498) represented its pooled remainder the best, while entry 22R (Δ_22R_ = 0.4902) differed the most from its complement.
- Based on the results of the genetic analysis and information regarding the current conservation status, 14 most appropriate wild populations for potential genetic reserve were identified. The used markers display a low level of genetic variation between the analyzed populations, and a split between Northern and Southern populations was observed.
- CWR species are essential genetic resources for plant breeding and food security. However, 11.5% of the European CWRs are threatened. Therefore, it is of utmost importance to determine their genetic compositions. These insights will provide the fundamental basis for making crucial decisions concerning future conservation strategies for *H. repens*.

## Graphical abstract

Fourteen most appropriate wild populations were successfully identified with SSRs, low level of genetic variation was estimated, and a split between Northern and Southern German populations was observed.

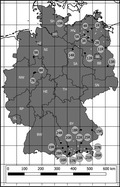

## 1 INTRODUCTION

Crop Wild Relative (CWR) species are, to some degree, related to the crops we use today. They often contain valuable resistance genes and other useful genetic traits and are thus essential resources for plant breeding (Hajjar & Hodgkin, 2007; Kole, 2011). When crossing the wild species with the crops, more resilient varieties can be bred (e.g., Diawara, Trumble, Quiros, & Millar, 1992; Martín‐Sánchez et al., 2003; Ochoa & Quiros, 1989; Paula, Dinato, Vigna, & Fávero, 2019; Simmons, Jarret, Cantrell, & Levi, 2019; Trumble, Derecks, Quiros, & Beier, 1990; Trumble, Diawara, & Quiros, 1998) which would contribute to broadening the breeding pool (Veteläinen & Nissilä, 2001).

The increase in the world's population, which is predicted to reach 10 billion in 2056 (United Nations, 2017), accompanied by a decrease in arable agricultural land (The World Bank Group, 2019) and forecast changes in climate, drives the needs of agriculture to enhance the productivity of crops (Henry, 2014; Shapter et al., 2013). However, finding the means to effect this enhancement is at risk. Of the 572 European CWRs assessed in a study by Bilz, IUCN Regional Office for Europe, and IUCN Species Survival Commission (2011), 11.5% are threatened (vulnerable to critically endangered) and for 29%, the available genetic data was insufficient (Bilz et al., 2011). The loss of these genetic resources will have unpredicted consequences for crop production and food security (Frese, Bönisch, Herden, Bönisch, Herden, Zander, & Friesen, 2018; Henry, 2014; Wehling, Scholz, Ruge‐Wehling, Hackauf, & Frese, 2017). There is, therefore, considerable interest in agricultural policies directed at protecting genetic resources in situ and ex situ (BMEL, 2015). Already, in the later 20th century, the signatory parties of the *International Treaty on Plant Genetic Resources for Food and Agriculture* and the *Convention on Biological Diversity* committed themselves to the protection of CWRs (CBD, 1992; FAO, 2001). The model and demonstration project “Genetische Erhaltungsgebiete für Wildsellerie (*Apium* und *Helosciadium*) als Bestandteil eines Bundesweiten Netzwerkes genetischer Erhaltungsgebiete in Deutschland‐ GE‐Sell” (Genetic Nature Reserves for Wild Celery (*Apium* and *Helosciadium*) as Part of a National Network in Germany) is one of the few projects, attempting to establish genetic reserves in practice (Frese, Bönisch, Herden, et al., 2018).

There are two main approaches to categorizing CWR in relation to their crops: The gene pool concept (Harlan & de Wet, 1971) and the taxon concept (Maxted, Ford‐Lloyd, Jury, Kell, & Scholten, 2006). The approach of Harlan and de Wet (1971) is based on crossability between the crop and the CWR and was applied in the above‐mentioned project (Frese, Bönisch, Herden, et al., 2018). In Germany, four wild celery species are considered to be CWR of *A. graveolens*: *A. graveolens* L. ssp. *graveolens*, *Helosciadium repens* (Jacq.) W.D.J. Koch, *Helosciadium inundatum* (Jacq.) W.D.J. Koch and *Helosciadium nodiflorum* (Jacq.) W.D.J. Koch. Pink et al. (1983) had no success in their attempt to cross *A. graveolens* crops with *H. nodiflorum*. There have as yet been no attempts at crossing the crop with *H. repens*. Since *H. repens* is closely related to *H. nodiflorum* (Ronse, Popper, Preston, & Watson, 2010), Frese, Bönisch, Herden, et al. (2018) advocated a temporary classification into the tertiary gene pool of *A. graveolens*. This gene pool represents the extreme outer limit of the potential gene pool of the crop (Harlan & de Wet, 1971).

*Helosciadium repens* belongs to the Apiaceae family. It is a small perennial herb which is widely distributed in Western and Southern Europe, parts of North Africa and the Canary Islands (Hultén & Fries, 1986; Muer, Sauerbier, & Cabrera, 2016; Ronse et al., 2010; Schoenfelder & Schoenfelder, 2012; Tutin, 1968). Despite its broad distribution area, the species is scarce and listed as near threatened in Europe (Bilz et al., 2011). It is also considered critically endangered in Germany classified with different levels of endangerment across the federal states (BfN, 2018a, 2018b). In Germany, the distribution area is divided roughly into two parts: The Northern region, which has the highest number of populations located in Mecklenburg‐West Pomerania (MV), and the Southern region, namely Bavaria (BY; BfN, 2018a). Even though *H. repens* has never been an abundant species in general (Burmeier & Jensen, 2008), its distribution area began to decline due to urbanization and changes in land‐use. This habitat shrinkage will continue in the future if model scenarios prove to be correct (Aguirre‐Gutiérrez, Treuren, Hoekstra, & Hintum, 2017; Burmeier & Jensen, 2009). The species is hemicryptophytic (Oberdorfer, 1983; Schubert & Vent, 1994). However, hydrophytic populations with their submerged hibernating organ can be found occasionally (Casper & Krausch, 1981; NLWKN, 2011; Schossau, 2000, cited in Hacker, Voigtländer, & Russow, 2003). It grows on alternating wet pastures, littoral zones of trenches and springs (Weber, 1995) and along slow running streams. Furthermore, populations growing in stagnant water can also be found.

This plant is a weak competitor against taller herbs or shrubs as it is light‐demanding and low‐growing. As a consequence, *H. repens* can often be found on mowed lawns at camping grounds, or areas with grazing management (Burmeier & Jensen, 2009; McDonalds & Lambrick, 2006). Due to its creeping stolon habitus, it occupies uncovered ground very quickly. However, even slight changes in grazing management which benefit its competitors can lead to drastic changes in population sizes (e.g., a shift of livestock or change in mowing periods). Should this be the case, populations can gradually disappear over several vegetation periods (Burmeier & Jensen, 2008, 2009; Naturschutzring Dümmer E.V., 2015 unpublished data). *Helosciadium repens* propagates not only clonally but also by seeds (Burmeier & Jensen, 2008; Hacker et al., 2003). It produces numerous self‐compatible flowers which produce nectar to attract small insects (East, 1940; Frank & Klotz, 1990; Ronse et al., 2010). From these monoicous, facultatively xenogamous flowers, two seeds are produced which have no mechanisms for long‐distance dispersal (Klotz, Kühn, & Durka, 2002; Lederbogen, 2000). However, endozoochoric propagation from birds is possible (Lederbogen, 2000). Additionally, the seeds can stay afloat for approximately 24 hr and are thus able to drift for at least short distances (Burmeier & Jensen, 2008). Dormant seeds build seed soil banks from which the species can recruit seedlings once there are gaps in the vegetation cover or less competition (Burmeier & Jensen, 2008).

The primary goal of this study is to find the most appropriate wild populations (MAWP) as candidates for genetic reserves of one of the CWR of *A. graveolens*: *H. repens*. The term MAWP was defined by S. Kell (Maxted et al., 2015) and describes an in situ conservation unit selected according to the proposed quality standards for genetic reserves of Iriondo et al. (2012).

A genetic reserve, as defined by Maxted, Hawkes, Ford‐Lloyd, and Williams (1997), is an area where the genetic diversity of natural populations is monitored and managed for long‐term conservation and captures as much of the genetic diversity of the target taxon as possible (Iriondo et al., 2012). For this, we characterized selected populations of *H. repens* in Germany with microsatellites (SSR). To understand the contribution of each population to the overall diversity within the entire set, we analyzed the genetic diversity and composition of 27 occurrences. Finally, MAWPs were chosen, using criteria based on the quality standards proposed by Iriondo et al. (2012). The required habitat, site, population, legal, social, and management data were recorded during the site visits. At the end of an eight‐step planning process (Frese, Bönisch, Herden, et al., 2018), we propose to establish genetic reserves for 14 MAWPs.

## 2 MATERIAL AND METHODS

### 2.1 Preselection of occurrences

A list of distribution data of *H. repens* in Germany was created with the help of database excerpts provided by the Landesumweltämter (environmental agencies, EA) and data from local botanical institutes. The heterogeneous data set was homogenized in order to make the records comparable. The inventory contained 1,040 entries, of which 78 populations were selected for a preliminary assessment. Populations were selected based on the following criteria. (a) The selection must include all kinds of habitats where the species was found. Therefore, populations were chosen from different eco‐geographic units of the second‐order (EGUs) according to Meynen and Schmithüsen (1959) to capture the genetic variation of adaptive traits. EGUs represent the regions with specific abiotic (climatic, geomorphologic, geologic, hydrologic, and soil conditions) and biotic features (flora and fauna). These geofactors can have considerable influence on the number and composition of secondary metabolites and on the organic compounds (Cirak et al., 2012; Forwick, Wunder, Wingender, Möseler, & Schnabl, 2003; Ramakrishna & Ravishankar, 2011; Szakiel, Pączkowski, & Henry, 2011; Zlatić & Stanković, 2017).

(b) In some cases, the data from the agencies included possible immediate threats in the comments field of the database excerpts. Those populations were not taken into account, as the risk of these becoming extinct in the near future was too high. (c) The populations should have at least a population size of 30 individuals. (d) Also, if possible, populations existing in nature reserves (NRs) were selected. These sites already provide the infrastructure that can be used to improve the conservation of the CWR target taxon. In comparison to areas without a conservation status, NRs can sustain a genetic reserve for a more extended period.

Permission from authorities and property owners was obtained. The sites were visited in the year 2015 in order to assess the suitability of the location and the conservation status of the occurrence. In some cases, in Bavaria and Mecklenburg‐West Pomerania, current monitoring data already existed and was used for further assessment. The collected data were stored in the GE‐Sell database available online at http://vm323.rz.uos.de/mapportal/pages/auswahl_gesell.php.

**Figure 1:**
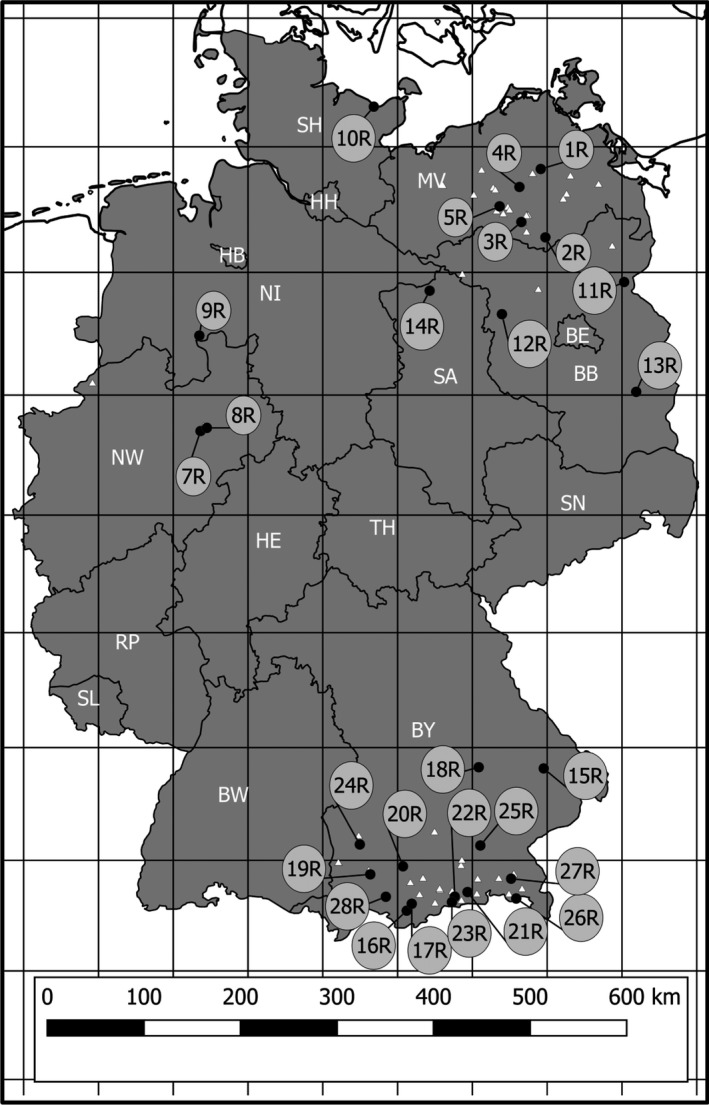
Provenance of the 27 analyzed German populations of *Helosciadium repens*. Black dots: analyzed populations; population IDs correspond with the Laboratory IDs in Table 1; white triangles: preliminary assessed and confirmed populations in 2015; white letters = Federal States of Germany (BB, Brandenburg; BE, Berlin; BW, Baden Wuerttemberg; BY, Bavaria; HB, Bremen; HE, Hesse; HH, Hamburg; MV, Mecklenburg–West Pomerania; NI, Lower Saxony; NW, North Rhine‐Westphalia; RP, Rheinland‐Pfalz; SA, Sachsen Anhalt; SH, Schleswig‐Holstein; SL, Saarland; SN, Saxony; TH, Thuringia); scale bar at equatorial scale; Pseudo‐Mercator Projection

The comparison of the first assessment with the date from the EAs indicated annual variations in population sizes. Locations with high population sizes were preferred to avoid traveling to sites with temporarily small population sizes. If the preliminary assessment in 2015, or the second visit in 2016 revealed immediate threats, the populations were not taken into account. Out of the confirmed occurrences, 27 populations were selected for sampling and genetic analysis in 2016 (Figure 1). The selected populations were located in Bavaria (BY; 15R‐ 28R), Brandenburg (BB; 11R‐ 13R), Mecklenburg‐West Pomerania (MV; 1R‐ 5R), Lower Saxony (NI; 9R), North Rhine‐Westfalia (NWR; 7R and 8R), Schleswig‐Holstein (SH; 10R), and Saxony‐Anhalt (ST; 14R).

### 2.2 Plant material and DNA extraction

Leaves from up to 30 individuals of 27 *H. repens* populations (Table 1) were collected (Brown & Marshall, 1995). If a population size was lower than 50 individuals, the number of sampled individuals was reduced (for the numbers of analyses samples see Table 3). Overall, 715 individuals were analyzed. The material was collected along a grid with a minimum distance of two meters, to avoid sampling from the same individual or plants with a high degree of kinship. The material was dried using silica gel and later used for the DNA isolation. Total genomic DNA was isolated using the InnuPREP Plant DNA Kit (Analytic Jena AG). As secondary metabolites inhibited the PCR, the protocol from the manufacturer was altered. After the incubation at 65°C for 30 min, 60 µl of Sorbent was added from the Diamond DNA Plant Kit (Diamond DNA), mixed on a shaker and centrifuged for 5 min on ca. 13,226 x *g*. If this action was performed after the final DNA elution, it resulted in the loss of the DNA (personal observation). The supernatant was then used in all further stages according to the instructions of the manufacturer. Sorbent is activated carbon with a high adsorption capacity. As it does not bind the DNA, it is therefore ideal for removing metabolites which potentially inhibit PCR reactions (for more information see the Federal Institute of Industrial Property, IPS Ru#1545641425588). Isolated DNA was diluted 1:20 and then used directly for PCR amplification.

**Table 1:** Provenance of 27 analyzed German populations of *Helosciadium repens* and arguments for or against a nomination as a most appropriate wild population

Laboratory ID	GE‐Sell ID	Location	EGU	Arguments for or against the nomination as MAWPs
1R	**MV‐GC‐20120912‐1400**	Mecklenburg‐ West Pomerania: Demmin, Lake Kummerow, Camping Ground	Rückland der mecklenburgischen Seenplatte)	The best representative in quality standards for a genetic reserve within the EGU; camping ground suits best for public relations
2R	MV‐WWR‐20150806‐1430	Mecklenburg‐ West Pomerania: Mecklenburg‐Strelitz, Wesenberg	Mecklenburgische Seenplatte	3R and 5R are the better representatives in quality standards for a genetic reserve within the EGU
3R	**MV‐GS‐20150928‐0930**	Mecklenburg‐ West Pomerania: Lake Müritz	Mecklenburgische Seenplatte	Biggest population in Mecklenburg‐ West Pomerania
4R	MV‐MSC‐20141030‐1400	Mecklenburg‐ West Pomerania: Lake Malchin	Rückland der mecklenburgischen Seenplatte	Small population; 1R is the best representative in quality standards for a genetic reserve within the EGU
5R	**MV‐DS‐20131029‐1030**	Mecklenburg‐ West Pomerania: Lake Müritz, Alt Schwerin	Mecklenburgische Seenplatte	The population is located in a wildlife reserve, already in focus of nature conservation foundation, which owns the site
7R	NRW‐LP‐20150819‐0945	North Rhine‐Westphalia: Soest, Lippstadt, Lake Margareten	Westfälische Tieflandbucht	Small area, 8R is the best representative in quality standards for a genetic reserve within the EGU
8R	**NRW‐DB‐20150818‐1831**	North Rhine‐Westphalia: Paderborn, Delbrück	Westfälische Tieflandbucht	The best representative within the EGU
9R	**NI‐OM‐20150812‐0955**	Lower Saxony: Diepholz, Hüde, Ochsenmoor	Dümmer‐Geestniederung	The only representative in this EGU; excellent collaboration with the local conservationists in charge; management plan already exist
10R	**SH‐TIV‐20150902‐0900/0910/0920**	Schleswig‐ Holstein: Plön, Blekendorf	Schleswig‐Holsteinisches Hügelland	Excellent collaboration to the local conservationists in charge; already in focus of nature conservation foundation, which owns the site
11R	Bbg‐PA‐20150723‐0702	Brandenburg: Barnim, Parsteinsee	Rückland der mecklenburgischen Seenplatte	Low vitality, 1R is the best representative in quality standards for a genetic reserve within the EGU
12R	**Bbg‐SE‐20150723‐1634**	Brandenburg: Seeblick	Elbtalniederung	Only representative in this EGU; high numbers of individuals
13R	**Bbg‐JE‐20150721‐1018**	Brandenburg: Dahme‐Spreewald, Schwielochsee	Ostbrandenburgisches Heide‐ und Seengebiet	Only representative in this EGU; large and vital population
14R	**ST‐KRAAT‐20130816‐1119**	Saxony‐Anhalt: Altmarkkreis Salzwedel, near Arendsee	Altmark	Only representative, excellent collaboration to the local conservationists in charge; the local group is highly interested
15R	BY‐DEG_FISC‐20151024‐1001	Bavaria: Deggendorf	Unterbayerisches Hügelland	Small population
16R	BY‐GAP_FARC‐20151021‐1004	Bavaria: Garmisch‐Partenkirchen, Farchant	Schwäbisch‐Oberbayerische Voralpen	Eutrophication, 22R is the best representative in quality standards for a genetic reserve within the EGU
17R	BY‐GAP_ESCH‐20160903‐1096	Bavaria: Garmisch‐Partenkirchen, Eschenlohe	Schwäbisch‐Oberbayerische Voralpen	Low vitality, small population, 22R is the best representative in quality standards for a genetic reserve within the EGU
18R	**BY‐KEH_NIED‐20150908‐1005**	Bavaria: Kelheim, Langquaid	Unterbayerisches Hügelland	Represents the whole composition of the analyzed populations the best
19R	BY‐KF_KAUF‐20150814‐1012	Bavaria: Kaufbeuren	Voralpines Hügel‐ und Moorland	Small population; eutrophication, 27R is the best representative in quality standards for a genetic reserve within the EGU
20R	BY‐LL_BISC‐20160828‐1022	Bavaria: Landsberg am Lech, Dießen am Ammersee	Voralpines Hügel‐ und Moorland	27R is the best representative in quality standards for a genetic reserve within the EGU
21R	BY‐MB_TRAC‐20150811‐1002	Bavaria: Miesbach, Fischbachau	Schwäbisch‐Oberbayerische Voralpen	Medium vitality, eutrophication, 22R is the best representative in quality standards for a genetic reserve within the EGU
22R	**BY‐MB_TRIN_20150802‐1003**	Bavaria: Miesbach, Kreuth	Schwäbisch‐Oberbayerische Voralpen	Represents the uniqueness population within this composition; large plain aquatic population
23R	BY‐MB_WILD‐20150731‐1001	Bavaria: Miesbach, Kreuth	Schwäbisch‐Oberbayerische Voralpen	Medium vitality, 22R is the best representative in quality standards for a genetic reserve within the EGU
24R	**BY‐MN_SALG‐20150804‐1019**	Bavaria: Unterallgäu, Salgen	Donau‐Iller‐Lech‐Platten	Single representative in this EGU; high numbers of individuals
25R	BY‐MUE_MARS‐20150829‐1027	Bavaria: Mühldorf a. Inn, Maitenbeth	Voralpines Hügel‐ und Moorland	Medium vitality, 27R is the best representative in quality standards for a genetic reserve within the EGU
26R	**BY‐TS_WINK‐20150812‐1014**	Bavaria: Traunstein, Reit im Winkl	Nördliche Kalkhochalpen	Single representative in this EGU; high numbers of individuals
27R	**BY‐TS_WINK‐20151114‐1001**	Bavaria: Traunstein, Übersee	Voralpines Hügel‐ und Moorland	A large population within a wildlife reserve
28R	BY‐WM_SAUW‐20160907‐1124	Bavaria: Weilheim‐Schongau, Prem	Voralpines Hügel‐ und Moorland	Small population, 27R is the best representative in quality standards for a genetic reserve within the EGU

Note

Laboratory ID = population work ID, GE‐Sell ID = reference IDs used in the project GE‐Sell (bold letters‐ MAWP), location = locations in Germany (Federal State: description of the location), EGU = eco‐geographic units (order two) based on Meynen and Schmithüsen (1959).

### 2.3 Primer design

The company TraitGenetics GmbH performed the design and construction of the forty‐nine genomic SSR primer, based on the sequenced nuclear genome of *H. repens*. All microsatellites were repeats of dinucleotides of various lengths. Forward primers of all sets were labeled with fluorescence dyes HEX or FAM (for primer sequences see Table 2). The markers were neutral and not subjected to any evolutionary constraint.

**Table 2:** SSR primers sets used in the analysis of 27 populations of *H. repens* in Germany, assessed with six microsatellites

Primer ID	Dye	F‐primer	R‐Primer
ANM0057	FAM	AATATTATTGATTGGAGTGCGTTT	TGAGGTTGTAATAGGCTATCATCAGT
ANM0066	HEX	TGGCAGCCTGGATAACTACC	AGTAAGGAGAAGTAACTGAACAAGAGA
ANM0077	HEX	AATACATACATACATGCCTTCACTAAG	CAATAAGTGCTTGAGAATCTAATAGG
ANM0079	HEX	AAGCCACATAGCAAACCTGC	CGTGCAAAGTTGTGGTGTCT
AXM0081	FAM	GGGAGTGATGGTAGGAGAGTAGAA	TGAGAATCAATTAATTTGGTGAAGG
AXM0083	FAM	TTGCCACTTTCATTACATCTTCA	AGAACATCCAAGTTATGCTGACAA
AXM0087	FAM	TCCAACCTAATCCATCTCTACACA	AAAGAGATACACAGTTATCGAGGAG
AXM0090	FAM	TCAAGATGGCCTTCTCAAGT	AAAGAAGGATACTGACCAGGCTT
AXM0091	FAM	ACGTAGAAACCTGCACCCAA	CCCTTTCTTTCTCCCTGATG
AXM0105	HEX	TCGTAGGGAGACCATGTAGCTT	AATGGGCCAACCCAAAGT
AXM0108	HEX	GCTAAATTTACGGTTGGTTCCTT	CTAATAGTTAACCCATAATTTGGAGAA

Note

Primer ID = identification code of the primer sets (bold letters‐ primer sets used in the final analysis), dye = fluorescence marker of the forward primer (HEX‐ Hexachloro‐Fluorescein, FAM‐ 5(6)‐Carboxyfluorescein), F‐primer = forward primer sequence, R‐primer = reverse primer sequence.

### 2.4 SSR amplification

A test sample set was designed based on three populations (1R, 2R, and 9R). From each population, ten individuals were used. Microsatellite amplification was carried out for all 49 primer sets in a final volume of 20 µl, containing 1 µl DMSO, 2 µl 10× reaction buffer B (Solis BioDyne), 1.6 µl MgCl_2_ (25 mM), 0.4 dNTP mix (10 mM), 0.6 µl of each primer (0.3 µM), 1 µl DNA, and 0.1 µl FIREPol Taq‐polymerase (Solis BioDyne). PCRs were carried out under the following touchdown PCR conditions for all loci: 94°5′30″, 56°45″, 72°1′ [94°30″, 55.5°45″, 72°1′]_6_ (lowering the annealing temperature by 0.5°C every cycle) [94°30″, 52°45″, 72°1′]_31_ 72°10′, 12°5′. Samples which failed in the first run were rerun using the 10 µl Biozym red HS Taq master mix (Biozym Scientific GmbH), 0.6 µl of each primer (0.3 µM), 1 µl DNA in a final volume of 20 µl. PCR products were checked on an agarose gel before being sent to TraitGenics for fragment analysis. The primer test revealed that eleven out of 49 SSR primer sets produce suitable products for further analysis. However, only six of these amplified regions across all populations successfully and were used in the further analysis. Individuals which failed to amplify in one or more primer sets were excluded.

The software Genemapper v5.0 (Thermal Fischer Scientific Inc.) was used to evaluate the chromatograms by identifying all microsatellite alleles and their respective sizes. Each call was checked manually and corrected if necessary. Primer sets which successfully amplified polymorphic products in all test populations were used to analyze all 27 populations.

### 2.5 Data analysis

Based on previous exclusion, out of the 763 collected individuals, 715 were used in the analysis (Table 3). SAS ProcAllele procedure was used to test the Hardy–Weinberg Principle (HWP) and calculate allele frequencies, polymorphic information index (PIC), observed (*H*_o_), and expected heterozygosity (*H*_e_) using 10^4^ permutations and 5,000 bootstraps pseudo‐replicates. The SSR data was converted manually into a genepop format and loaded in R using the package *adegenet* for further analyses (Jombart & Collins, 2015). Private alleles (alleles unique to a specific population) were counted with the function *private_alleles* from the R package *poppr*2.8.1 (Kamvar, Tabima, & Grünwald, 2014), and rare alleles, at a frequency ≤ 0.05, were recovered from the SAS output data. Rare and private alleles were related to the sample size of the population. Allelic richness was measured with rarefaction using the *allel.rich* function from the R package *PopGenReport* (Gruber & Adamack, 2014) and based on the works of Hurlbert (1971). The smallest number of individuals sampled across all combinations of populations and loci was 14. The measure of deviation from panmixia at the local scale (*F*_IS_) was calculated with the software Fstat2.9.3.2 (Goudet, 2001) and the fixation index F with GenAlEx6.51b2 (Peakall & Smouse, 2006, 2012). Tests for significance were carried out with the *geom_signif* function using the R package *ggplot2*. Plots and graphs were drawn using the function *ggplot* from the R package *ggplot2* (Wickham, 2016).

**Table 3:** Genetic diversity parameters for each of the 27 analyzed German populations of *Helosciadium repens* assessed with six microsatellites

Laboratory ID	*n*	MLG	SLG	A	Rare	Private	all.rich	Mean Ho	Mean He	*F*	*F*_IS_	delta*SD*	Cat	Form
1R	27	11	20	16	0	0.037	1.870	0.093	0.167	**0.336**	**0.462**	0.3884	N	terr
2R	28	3	18	14	0	0	1.857	0.250	0.253	−0.009	0.028	0.36	N	terr
3R	29	8	11	11	0	0	1.596	0.201	0.171	**0.087**	−0.163	0.375	N	terr
4R	**7**	7	15	12	0	0	1.910	0.214	0.274	**0.203**	**0.289**	0.3136	N	terr
5R	25	3	9	11	0	0	1.251	0.013	0.039	**0.656**	**0.667**	0.447	N	terr
7R	26	4	8	8	0	0	1.116	0.006	0.019	**0.490**	**0.667**	0.3064	N	terr
8R	30	7	11	11	0.033	0	1.489	0.089	0.094	**0.143**	**0.066**	0.4104	N	terr
9R	30	6	12	12	0	0	1.568	0.106	0.130	**0.330**	**0.206**	0.2798	N	terr
10R	29	6	7	7	0	0.034	1.109	0.023	0.021	−0.074	−0.057	0.3798	N	terr
11R	30	4	10	10	0	0.033	1.176	0.028	0.027	−0.026	−0.01	0.2952	N	terr
12R	26	3	8	8	0	0	1.140	0.026	0.024	−0.040	−0.031	0.3409	N	terr
13R	30	1	6	6	0	0	1.000	0.000	**0.**000		**NA**	0.4394	N	terr
14R	15	5	10	11	0	0.200	1.471	0.044	0.083	**0.470**	**0.491**	0.3598	N	terr
15R	29	27	31	19	0.034	0	2.662	0.448	0.501	**0.095**	**0.122**	0.2829	S	terr
16R	30	5	10	9	0	0	1.443	0.178	0.155	**0.137**	−0.13	0.3155	S	aqu
17R	28	23	22	16	0	0	2.177	0.274	0.352	**0.211**	**0.24**	0.3914	S	terr
18R	27	22	18	17	0.037	0	2.051	0.321	0.310	−0.012	−0.016	0.2498	S	terr
19R	30	5	9	10	0	0	1.631	0.383	0.260	−0.241	−0.464	0.3617	S	aqu
20R	29	6	10	10	0	0	1.409	0.172	0.178	**0.013**	**0.047**	0.2966	S	aqu
21R	30	10	14	13	0.067	0	1.920	0.339	0.284	−0.184	−0.177	0.4754	S	aqu
22R	29	12	15	14	0	0.034	1.784	0.247	0.266	**0.051**	**0.089**	0.4902	S	aqu
23R	30	2	7	7	0	0	1.037	0.006	0.005	−0.017	0	0.4161	S	aqu
24R	29	8	11	12	0	0	1.515	0.310	0.191	−0.329	−0.617	0.3136	S	aqu
25R	28	7	12	13	0	0	1.868	0.512	0.301	−0.505	−0.69	0.3845	S	aqu
26R	29	29	36	21	0	0.069	3.152	0.420	0.586	**0.248**	**0.301**	0.342	S	terr
27R	26	20	30	18	0.038	0	2.599	0.340	0.439	**0.350**	**0.245**	0.268	S	terr
28R	9	8	14	14	0	0.333	2.068	0.296	0.320	**0.094**	**0.132**	0.3732	S	terr

Note

Laboratory ID = population work IDs corresponding with the Laboratory IDs in Table 1 (bold = MAWPs), *n* = sample size in the analysis, MLG = numbers of multi‐locus genotypes, SLG = numbers of single‐locus genotypes, A = numbers of alleles, rare = number of rare alleles per individual, private = number of rare alleles per individual, all.rich = average allelic richness, mean *H*_o_ = mean observed heterozygosity, mean *H*_e_ = mean expected heterozygosity, *F* = Fixation Index (bold‐ positive values), *F*_IS_ = *F*_IS_ Index after Weir and Cockerham (1984) (bold‐ positive values), delta*SD* = compositional differentiation at genotype level, cat = category (S = Southern populations, *N* = Northern populations), form = ecological form (terr = terrestrial; aqu = aquatic).

The measure Δ is free of model assumptions such as the presence of large, random mating populations in the Hardy–Weinberg equilibrium (HWE; Gregorius, Gillet, & Ziehe, 2003) and ranges between 0 (no genetic distance between a pair of populations) and 1 (highest possible genetic distance between a pair of populations). The software DifferInt was used to calculate the complementary compositional differentiation among populations, whereby Δ*_j_* is the contribution of the *j*th population to genetic differentiation. Δ*_j_* is the genetic distance of the *j*th population to the pooled remainder (“the complement”). A population with Δ*_j_* = 0 population represents exactly its complement, while the genetic composition of a population with Δ*_j_* = 1 is entirely different from its complement. Δ*_SD_* quantifies the average degree to which all populations differ from their complements (Gillet, 2013). DifferInt calculates the complementary compositional differentiation at different levels of genetic integration: single‐locus genotypes (SLG) and the multi‐locus genotypes (MLG). Effects of differences among the populations' gene pools and gene association within the gene pools on differentiation were compared by two permutation analysis (Gillet, 2013; 10^3^ random permutations).

Population structure analysis was carried out using a discriminant analysis of principal components (DAPC) implemented in the R package *adegenet* (Jombart, Devillard, & Balloux, 2010). This analysis is comparable with an analysis by the software *Structure* (Evanno, Regnaut, & Goudet, 2005). However, it does not assume random mating populations in HWE (Jombart et al., 2010). The function *find.clusters* was used to identify the number of genetic groups (hereafter *K*; 50,000 iterations and five random starting centroids) and the function *optim.a.score* to find the optimal number of principal components. Additionally, another independent nonmodel approach was used to confirm the result. This method was based on the replicated nonhierarchical *K*‐means clustering (Hartigan & Wong, 1979) using the R‐script of Arrigo et al. (2010). We performed 5 × 10^4^ independent runs (starting from random points) for each of the assumed groups between two and 30. The intergroup inertia was recorded as a proxy of clustering accuracy, and the delta *K* values were calculated (Evanno et al., 2005) using the method adopted by Arrigo et al. (2010). The values with the highest delta *K* were considered the optimal number of groups in the data. Pie charts showed the percentage of individuals assigned to a genetic group. They were drawn using the function *pie* from the R package *graphics* (Becker, Chambers, & Wilks, 1988; Cleveland, 1994). All packages were used in RStudio 1.0.153 (R Core Team, 2017; RStudio Team, 2016).

Maps were drawn with QGIS‐2.8.1‐Wien (QGIS Development Team, 2009) with a pseudo‐Mercator projection. Natural Earth (http://www.naturalearthdata.com) provided the free vector and raster map data.

### 2.6 Selection criteria for MAWPs

The results from DifferInt were used to guide the selection of populations for genetic reserves. As means for conservation are always limited, the procedure was started with the population which had the lowest and highest Δ*_j_* at the gene pool level. (a) In every EGU represented in the set of 27 sites, at least one genetic reserve should be established to maximize the chance of capturing adaptive trait variation. To this end, one population was selected from each EGU. (b) Large population size was preferred over smaller population size. (c) As genetic reserve management relies on the support of local nature conservation agencies, other institutional stakeholders and volunteers, organizational and social aspects were also taken into account. (d) If the collectors found an immediate threat during the collection phase in 2016, the population in question was not considered as a MAWP. (e) Populations with an existing management plan, regardless of their conservation status, were given priority.

## 3 RESULTS

### 3.1 Distribution

Of the 78 preliminary assessed sites, 59 contained *H. repens* populations. The largest population in MV was 3R with a distribution area exceeding 12,000 m^2^. *Helosciadium repens* is often found in patches rather than in continuous populations. Considering this, 12R with 400 m^2^ of a populated area was the largest population in the whole Northern area. In BY, the largest population was 22R with a population area of 350 m^2^, distributed over an area of 89,000 m^2^.

### 3.2 SSR analysis

**Figure 2:**
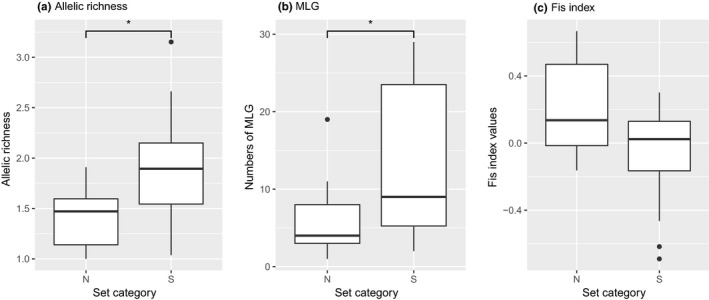
Comparison of the 27 analyzed German populations of *Helosciadium repens* assessed with six microsatellites. (a) Allelic richness (b) multi‐locus genotype (MLG) (c) *F*_IS_ Index values. N: northern populations (1R–14R), S: southern populations (15R–28R), asterisks indicate significance at the 0.05 level

The numbers of alleles per locus ranged from four to nine (AXM0105 and AXM0081, respectively), and the numbers of alleles per population ranged from six to 21 (13R and 26R, respectively). The PIC ranged between 0.3646 (AXM0105) and 0.5802 (AXM0090). Out of the 38 distinct alleles, 12 alleles were private and three were rare (Table 3). The H_o_ and the H_e_ of each locus ranged from 0.1748 to 0.2755 (AXM0087 and AXM0105) and 0.2756 to 0.6389 (AXM0087 and AXM090), respectively. Twenty‐two populations were not in the HWE (*p* < .05; Table S1). From the 162 cases (six primer sets × 27 populations), a significant deviation from the HWE was found in 49, and in 54 cases the markers were monomorphic. The only populations that were in HWE were 10R, 11R, 12R, 13R, and 23R (Table S1). In these populations, one to three markers had heterozygote genotypes and in 13R all the markers were homozygote. The *F*_IS_ Index ranged from −0.617 (24R) to 0.667 (5R and 7R; Table 3). Out of the 27 occurrences, ten showed an excess of heterozygosity, while 15 showed an excess of homozygosity (Table 3, excess of homozygosity in bold in the *F*_IS_ column). According to the *F*_IS_ Index, population 23R showed panmixia (Table 3). The fixation index *F* varied between −0.505 (25R) and 0.656 (5R). Out of the 27 populations, 16 exhibited inbreeding (Table 3). Ten populations showed an excess of heterozygosity (Table 3, excess of homozygosity in bold in the F column). The allelic richness and the amount of MLGs were significantly higher among the BY populations (S) in comparison to the Northern populations (N) (*p* < .05; Figure 2, Table 3). However, the amount of SLG, rare, and private alleles and the *F*_IS_ Index values were not significantly different (data not shown).

### 3.3 Complementary compositional differentiation

The numbers of SLG spanned from eight to 16 per locus (ANM0079 and AXM0105 with the lowest and AXM0090 with the highest count) and ranged from six to 36 (13R and 26R, respectively) in populations. The MLG spanned from one to 29 (13R and 23R with the lowest and 26R with the highest count). Within the whole data set (715 individuals and six markers), 68 SLG and 235 MLG were identified. Within populations, some MLGs were found to be duplicated ranging from two to 30 times. Population 13R was composed of only one MLG (Table 3).

The mean compositional differentiation at the genotype level was ∆*_SD_* = 0.3455 and increased to ∆*_SD_* = 0.3598 at mean SLG and ∆*_SD_* = 0.3691 at the MLG level. At the mean SLG and the MLG level, the ∆*_SD_*‐values observed were higher than 95% of all ∆*_SD_*‐values generated by the first permutation analysis. At all levels of integration, the ∆*_SD_*‐values were higher than 95% of all ∆*_SD_*‐values generated by second permutation analysis.

**Figure 3:**
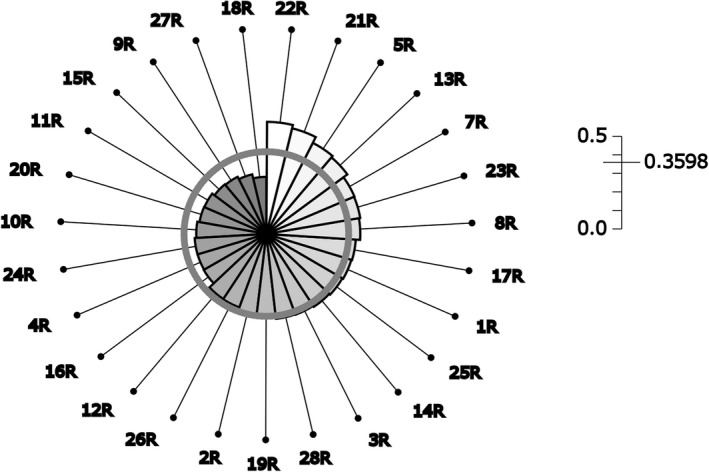
Snail diagram showing the differentiation of each of the 27 analyzed German populations of *Helosciadium repens* to their complement at the gene pool level. The data were generated with six microsatellites and estimated by the software DifferInt. The side length of a sector quantifies the contribution of each occurrence to the differentiation. The gray circumference represents the overall ∆*_SD_* values, which are given at the top right of the chart. Populations ID correspond with the Laboratory IDs in Table 1

22R was identified as the population with the highest ∆*_SD_*. Thus, it represented the population which differs most from the complement. The population 18R with the lowest ∆*_SD_* was the population which represents the whole complement the best (Figure 3, Table 3).

### 3.4 Discriminant analysis of principal components

The Bayesian information criterion (BIC) versus number‐of‐clusters plot showed no clear indication of the “true *K*” (data not shown). In the ∆*K* versus numbers of groups (*K*) plot, the value with the highest ∆*K* was at *K* = 2. However, a *K* between two and six was also considered possible (Figure S1). Therefore, we performed the DAPC with *K* equals two, four, and six. Populations were associated with the cluster with the highest obtained cluster assignment.

**Figure 4:**
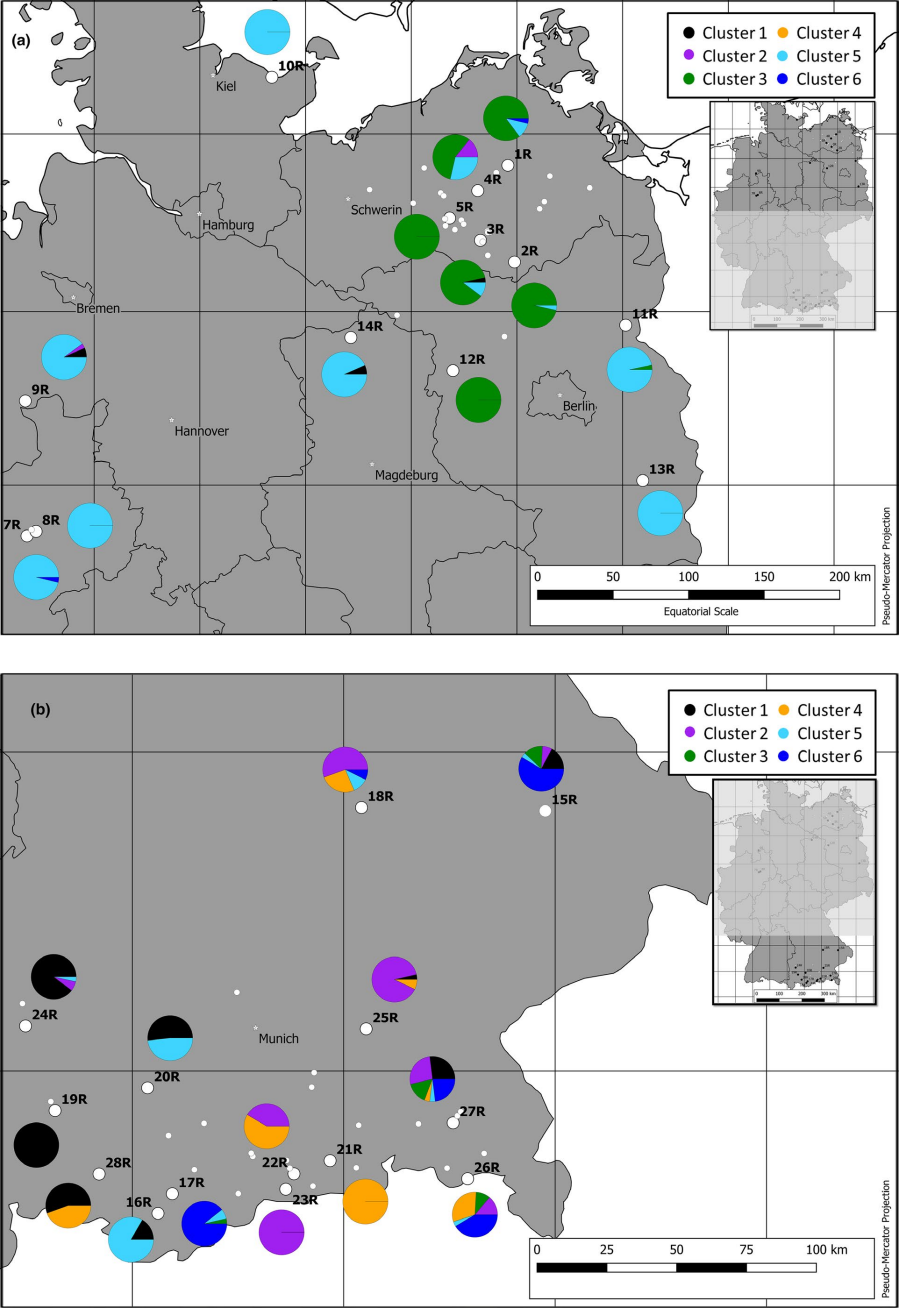
Discriminant Analysis of Principal Components (DAPC) with *K* = 6 clusters of all 27 analyzed German populations of *Helosciadium repens* based on the results of six microsatellites. (a) Northern part of Germany; (b) Southern part of Germany. Pie charts showing the percentage of individuals assigned to a cluster; white dots‐analyzed populations, populations ID correspond with the Laboratory IDs in Table 1; small white dots‐preliminarily assessed populations in 2015

For *K* = 2 the DAPC showed a division of N and S populations (data not shown). Only one population (16R from BY) did not coincide with its geographical distribution (with 83% of the individuals affiliated with the Northern cluster). Three populations (1R, 3R, and 4R) also had some individuals (<14%) affiliated with the Southern cluster. In the Southern cluster, there were eight populations with individuals associated with the Northern cluster (between 3% and 48%). For *K* = 4 and *K* = 6, the DAPC revealed similar, but more detailed clustering, compared with *K* = 2 (data for *K* = 4 not shown). However, with *K* = 6, only one population did not coincide with its geographical distribution (16R). Therefore, *K* = 6 was regarded to be the optimal number of clusters (Figure 4; for exact numbers, see Table S2).

Most clusters can be correlated with specific geographical regions. Populations from MV (1R, 2R, 3R, 4R, and 5R) and Western BB (12R) were allocated explicitly to cluster three. The rest of the North German populations were mostly linked to cluster five. Some individuals in a population were not assigned to the same cluster as the rest of the population (7% on average). When they are compared to the N populations, the S populations are more heterogeneous. Nevertheless, some populations from a specific region were allocated to a particular cluster (such as Western Bavaria populations—19R, 20R, 24R, and 28R to cluster one and the central and Southern populations—18R, 23R, and 25R to cluster two) the regions which were affiliated to specific clusters were mostly overlapping (28% on average). Populations 15R, 18R, 20R, 22R, 26R, 27R, and 28R retrieved relatively high affiliation with more than one cluster. The BY populations can be organized into three groups according to the cluster assignment: East BY with 15R, 26R 27R, central BY with 18R, 21R, 22R, 23R, 25R, and West‐BY with 19R, 20R, 24R, 28R. Occurrences 16R and 20R had a high affiliation to cluster five, and 17R to cluster six. There was no correlation between the clusters and EGUs.

### 3.5 Selection of MAWPs

Besides the two selected populations based on the results from DifferInt (22R and 18R), populations 1R, 3R, and 5R from MV, 8R from east Muensterland region, 9R from Lower Saxony (NI), 12R and 13R from Brandenburg (BB), and 24R, 26R, and 27R from BY were also selected as MAWPs (for justification see Table 1). Additionally, two populations were selected as complementary though suboptimal MAWPs. These were the only representatives of their EGU but had a critically low population size (14R from Saxony‐Anhalt‐ ST) or was introduced (10R from Schleswig‐Holstein‐ SH).

## 4 DISCUSSION

Our study presents an analysis of genetic diversity and genetic differentiation based on a set of populations of *H. repens* sampled within the entire distribution area in Germany. The three main results derived from the analysis of 27 occurrences with six SSR markers are the following: (a) the analyzed markers show a low level of genetic variation between populations in Germany. (b) The populations are divided into Northern and Southern populations. (c) MAWPs suited to establish genetic reserves were identified and recommended.

### 4.1 Low level of genetic variation

The first permutation analysis randomly permutes the alleles among the individuals within each population. In a panmictic population, one would expect that gene association in individuals do this independent of the allelic type at each locus and type at a given level of integration (Gillet, 2013). If this hypothesis were correct, the ∆*_SD_*‐values of the integration level SLG and MLG would be within the 95% confidence interval of all ∆*_SD_*‐values generated by the first permutation analysis (Gillet, 2013). However, from the data generated by the SSR analysis, this hypothesis must be rejected. Tests for HWP also indicated nonrandom mating in 22 of the analyzed populations (Table S1).

One explanation for the indication of nonrandom mating revealed by the markers could be explained with runner growth. It is namely *H. repens* primary strategy to colonize open areas. However, the method of collecting material was designed to avoid sampling from the same individual or plants with a high degree of kinship. Another, and yet more likely explanation would be self‐fertilization or preferential mating within half‐ or full‐sib families. The high number of MLG duplications within populations and the excess of homozygotes shown in 14 populations by the *F*_IS_ and F‐Index seem to confirm this interpretation (Table 3). *Helosciadium repens* does produce high amounts of seeds. A prime example was population 13R, which is composed of only one MLG. As 80% of the individuals observed in 2016 were flowering, it is probably not a clonal population.

In 12 populations either the *F*_IS_ or F‐Index, or both values, were negative (Table 3). Small populations, or low numbers of reproducible individuals, overdominance, self‐incompatibility (SI) or asexual propagation are common explanations (Stoeckel et al., 2006). As the markers used were neutral and *H. repens* is not known for possessing any self‐incompatibility systems, the most likely explanation would be asexual reproduction. Almost all aquatic populations were among those 12 cases (except 22R). Schossau (2000, cited in Hacker et al., 2003) said that aquatic and semi‐aquatic populations tend to prefer vegetative growth. Nearly, all aquatic occurrences tend not to produce flowers. However, our study did not find any significant difference in the allelic richness or the mean ∆*_SD_*‐values between aquatic and terrestrial populations (Table 3).

The second permutation analysis randomly permutes the individuals with their genetic types among the populations. The forces that associate individuals with populations do this independently of their genetic type at a given level of integration if the observed ∆*_SD_*‐values are within a 95% confidence interval (Gillet, 2013). This hypothesis must be rejected due to differences among the gene pools of the 27 occurrences that were not randomly distributed. In other words, there is possibly no migration between the populations.

### 4.2 A North‐South split of the German distribution area

The comparison of the allelic richness and MLG between the North and the South revealed that S populations tend to be more diverse (Figure 2). This distinction is also visible in the DAPC map (Figure 4). The S populations (mostly the South‐Eastern) are part of various genetic clusters compared with the N populations. One plausible explanation for this difference in diversity can be given by assuming that *H. repens* refugia during multiple glacial periods was somewhere in the South of Europe (possibly South‐East). Spalik, Banasiak, Feist, and Downie (2014) estimated that *H. repens* diverged approximately two million years ago and, therefore, has been influenced by glacial and interglacial periods. During the recolonization of the Northern parts after the last glacial maximum, diversity was lost due to bottleneck effects (Hewitt, 1996, 1999). Similar events are also known for *Calluna vulgaris* (Mahy, Vekemans, Jacquemart, & Sloover, 1997), various Bryophytes (Cronberg, 2000), *Abies alba* (Konnert & Bergmann, 1995), *Allium ursinum* (Herden, Neuffer, & Friesen, 2012) and many other European species. To prove this assumption, a broader study on a European scale would be necessary.

### 4.3 Successfully identified MAWPs

Candidates for potential genetic reserves were successfully identified using SSR markers, previously collected populations and site data. With the lowest ∆*_SD_* value, the population in BY, Kellheim (18R) resembles the genetic diversity of all remaining 26 populations better than any other (Figure 3, Table 3). The population from BY, Miesbach (22R) had the highest ∆*_SD_* value, which means it differed the most from its complement. One can interpret this high differentiation as specific adaptation to this site. The microsatellites are well suited to obtaining insight into genetic variation, but they cannot detect adaptive trait variations. Therefore, we increased the chance to capture adaptive trait variations by also choosing populations from different EGUs. The 27 populations were present in 13 different EGUs. The current selection of the MAWPs had representatives in all 13 EGUs (Table 1). A large population size increases the chance of sustaining long‐term population viability and is one of the key quality standards proposed by Iriondo et al. (2012). The largest Northern populations based on distribution over a specified area (MV, Großer Schwerin‐ 3R) and the largest occupied area (BB, Seeblick‐ 12R) were included. The population in BY, Miesbach (22R) was also included as constituent part of the MAWP candidates because to its size, and due to the fact that it is the largest analyzed population in Southern Germany. At the time of determining the areas the taxonomical status both forms take (aquatic and terrestrial) was still not clear. If both had been mentioned in the same source, the authors have always addressed them independently (Casper & Krausch, 1981; Hacker et al., 2003; NLWKN, 2011; Voightländer & Mohr, 2008). Therefore, the set of MAWPs from BY also include two aquatic populations, which represent 40% of the BY candidates. Recently, Herden and Friesen (2019) compared both forms genetically and morphologically and found no evidence for taxonomic division.

Due to limited funding, the selection of MAWPs also needs to be centered on feasibility and cost‐effectiveness. Naidoo et al. (2006) pointed out the importance of economic costs in conservation projects. By prioritizing sites on already protected areas and areas with substantial support from local organizations (governmental or nongovernmental), the acquisition and management costs (Naidoo et al., 2006) were minimized. Management plans and facilities already exist in NRs and may only need to be changed slightly for the benefit of the target taxon. Also, the long‐term persistence of a genetic reserve within protected areas is far more likely due to the laws and regulations to which they are subject. As a genetic reserve has no legal power and is extremely dependent upon volunteer work, social aspects (such as the interests of the landowners) are considerably important, and scientific reasoning has to take second place. However, rejection of a particular population does not mean that they are irrelevant or too insignificant to be included in future studies.

## 5 CONCLUSIONS

Our study showed that the eight‐step process proposed by Frese, Bönisch, Herden, et al. (2018) (for an English version see also Frese, Bönisch, Nachtigall, Bönisch, Nachtigall, & Schirmak, 2018) is well suited for identifying MAWPs for establishing genetic reserves. Based on this study, the first European genetic reserves for *H. repens* were established in June 2019 (3R and 12R). In Germany, the genetic reserve has no legal status. Long‐term success is highly dependent on the support and active collaboration of local people. *Helosciadium repens* patchy population structure should be considered when collecting seeds for storage in gene banks. Seeds from every MAWP should be collected for ex situ preservation of genetic diversity in gene banks. We recommend making the samples available for plant breeders and conservationists, as the sustainable use of wild populations is an argument toward investing in further conservation activities. The seeds can be stored in the WEL Gene Bank (National Gene Bank for German Crop Wild Relative Species, Botanical Garden of Osnabrueck, Germany; see Table 1 for reverence IDs).

## CONFLICT OF INTEREST

None to declare.

## AUTHORS' CONTRIBUTION

N.F. and T.H. conceived the ideas. T.H. homogenized the data excerpts did the laboratory work and led the writing of the manuscript. M.B. organized the data excerpts, managed the first and second assessment of the sites, and together with T.H. was involved in the decision process of the MAWPs. All authors contributed critically to the draft.

### Open Research Badges

This article has been awarded Open Data Badge for making publicly available the digitally‐shareable data necessary to reproduce the reported results. The data is available at https://doi.org/10.5061/dryad.rr4xgxd5c.

## Data Availability

The data are available under https://doi.org/10.5061/dryad.rr4xgxd5c.
