# Supplementary figures and images for: Genetic diversity of Helosciadium repens (Jacq.) W.D.J. Koch (Apiaceae) in Germany, a Crop Wild Relative of celery
Source: Ecol Evol. 2019 Dec 17;10(2):875–90. doi: 10.1002/ece3.5947 (PMC6988547; doi:10.1002/ece3.5947)

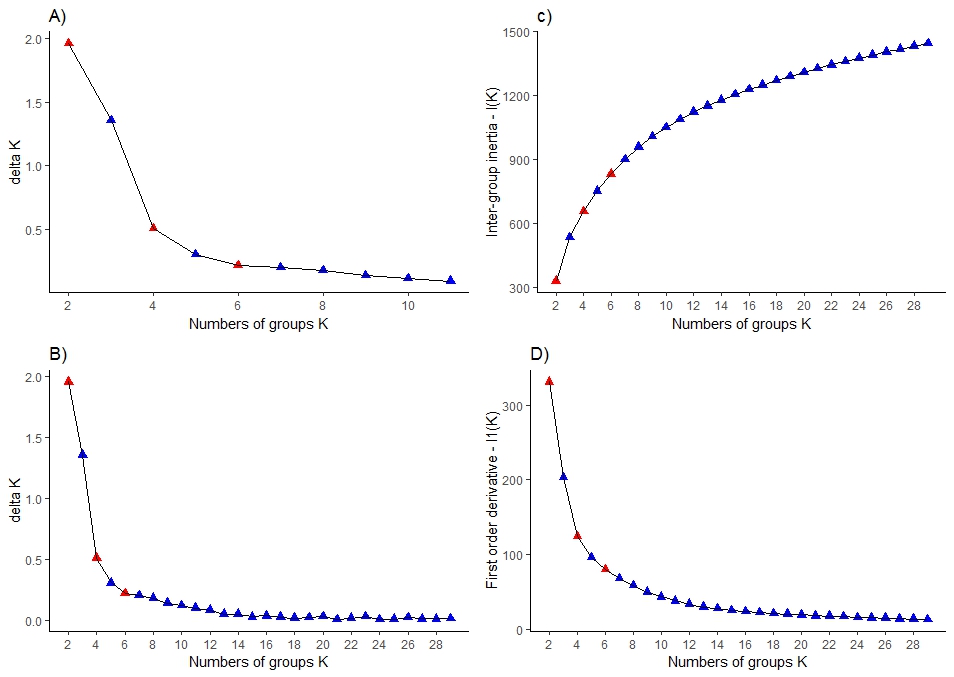

Supplement: Supplementary file 1 [file ECE3-10-875-s001.jpeg]
