# Supplementary material for: Genetic diversity of Helosciadium repens (Jacq.) W.D.J. Koch (Apiaceae) in Germany, a Crop Wild Relative of celery
Source: Ecol Evol. 2019 Dec 17;10(2):875–90. doi: 10.1002/ece3.5947 (PMC6988547; doi:10.1002/ece3.5947)
Supplement: Supplementary file 2 [file ECE3-10-875-s002.docx]

Table S1: Statistical parameters from 27 populations of *Helosciadium repens* in Germany assessed with six microsatellites.

|  |  | **Locus** | | | | | |
| --- | --- | --- | --- | --- | --- | --- | --- |
| **Lab ID** | **Parameter** | **P79** | **P81** | **P83** | **P87** | **P90** | **P105** |
| 1R | A | 3 | 3 | 2 | 1 | 4 | 3 |
|  | Ho | 0,15 | 0,07 | 0,04 | 0,00 | 0,19 | 0,11 |
|  | He | 0,14 | 0,07 | 0,10 | 0,00 | 0,32 | 0,36 |
|  | Chi^2 | 0,173 | 0,040 | 11,304 | 0,000 | 33,567 | 22,537 |
|  | DF | 3 | 3 | 1 | 0 | 6 | 3 |
|  | Pr(chi^2 >) | 0,982 | 0,998 | 0,001 | 1,000 | 0,000 | 0,000 |
|  | Pr.exact | 1 | 1 | 0,25 | 1 | 0 | 0 |
| 2R | A | 3 | 1 | 4 | 2 | 2 | 2 |
|  | Ho | 0,25 | 0,00 | 0,46 | 0,07 | 0,43 | 0,29 |
|  | He | 0,32 | 0,00 | 0,50 | 0,07 | 0,38 | 0,24 |
|  | Chi^2 | 2,171 | 0,000 | 2,283 | 0,038 | 0,571 | 0,778 |
|  | DF | 3 | 0 | 6 | 1 | 1 | 1 |
|  | Pr(chi^2 >) | 0,538 | 1,000 | 0,892 | 0,845 | 0,450 | 0,378 |
|  | Pr.exact | 0,25 | 1 | 0,5 | 1 | 0,75 | 1 |
| 3R | A | 3 | 3 | 1 | 1 | 2 | 1 |
|  | Ho | 0,97 | 0,07 | 0,00 | 0,00 | 0,17 | 0,00 |
|  | He | 0,59 | 0,13 | 0,00 | 0,00 | 0,31 | 0,00 |
|  | Chi^2 | 29,460 | 58,000 | 0,000 | 0,000 | 5,591 | 0,000 |
|  | DF | 3 | 3 | 0 | 0 | 1 | 0 |
|  | Pr(chi^2 >) | 0,000 | 0,000 | 1,000 | 1,000 | 0,018 | 1,000 |
|  | Pr.exact | 0 | 0 | 1 | 1 | 0 | 1 |
| 4R | A | 2 | 2 | 1 | 2 | 3 | 2 |
|  | Ho | 0,57 | 0,00 | 0,00 | 0,14 | 0,43 | 0,14 |
|  | He | 0,49 | 0,24 | 0,00 | 0,13 | 0,64 | 0,13 |
|  | Chi^2 | 0,194 | 7,000 | 0,000 | 0,041 | 3,142 | 0,041 |
|  | DF | 1 | 1 | 0 | 1 | 3 | 1 |
|  | Pr(chi^2 >) | 0,659 | 0,008 | 1,000 | 0,839 | 0,370 | 0,839 |
|  | Pr.exact | 1 | 0 | 1 | 1 | 0,25 | 1 |
| 5R | A | 3 | 1 | 1 | 2 | 3 | 1 |
|  | Ho | 0,04 | 0,00 | 0,00 | 0,00 | 0,04 | 0,00 |
|  | He | 0,08 | 0,00 | 0,00 | 0,08 | 0,08 | 0,00 |
|  | Chi^2 | 50,000 | 0,000 | 0,000 | 25,000 | 50,000 | 0,000 |
|  | DF | 3 | 0 | 0 | 1 | 3 | 0 |
|  | Pr(chi^2 >) | 0,000 | 1,000 | 1,000 | 0,000 | 0,000 | 1,000 |
|  | Pr.exact | 0 | 1 | 1 | 0 | 0,25 | 1 |
| 7R | A | 2 | 1 | 1 | 1 | 2 | 1 |
|  | Ho | 0,04 | 0,00 | 0,00 | 0,00 | 0,00 | 0,00 |
|  | He | 0,04 | 0,00 | 0,00 | 0,00 | 0,07 | 0,00 |
|  | Chi^2 | 0,010 | 0,000 | 0,000 | 0,000 | 26,000 | 0,000 |
|  | DF | 1 | 0 | 0 | 0 | 1 | 0 |
|  | Pr(chi^2 >) | 0,920 | 1,000 | 1,000 | 1,000 | 0,000 | 1,000 |
|  | Pr.exact | 1 | 1 | 1 | 1 | 0 | 1 |
| 8R | A | 2 | 2 | 2 | 2 | 1 | 2 |
|  | Ho | 0,00 | 0,13 | 0,10 | 0,17 | 0,00 | 0,13 |
|  | He | 0,06 | 0,12 | 0,10 | 0,15 | 0,00 | 0,12 |
|  | Chi^2 | 30,000 | 0,153 | 0,083 | 0,248 | 0,000 | 0,153 |
|  | DF | 1 | 1 | 1 | 1 | 0 | 1 |
|  | Pr(chi^2 >) | 0,000 | 0,696 | 0,773 | 0,619 | 1,000 | 0,696 |
|  | Pr.exact | 0,25 | 1 | 1 | 1 | 1 | 1 |
| 9R | A | 1 | 2 | 2 | 2 | 2 | 3 |
|  | Ho | 0,00 | 0,40 | 0,03 | 0,00 | 0,00 | 0,20 |
|  | He | 0,00 | 0,32 | 0,03 | 0,18 | 0,06 | 0,18 |
|  | Chi^2 | 0,000 | 1,875 | 0,009 | 30,000 | 30,000 | 0,370 |
|  | DF | 0 | 1 | 1 | 1 | 1 | 3 |
|  | Pr(chi^2 >) | 1,000 | 0,171 | 0,926 | 0,000 | 0,000 | 0,946 |
|  | Pr.exact | 1 | 0,5 | 1 | 0 | 0 | 1 |
| 10R | A | 1 | 2 | 1 | 1 | 1 | 1 |
|  | Ho | 0,00 | 0,14 | 0,00 | 0,00 | 0,00 | 0,00 |
|  | He | 0,00 | 0,13 | 0,00 | 0,00 | 0,00 | 0,00 |
|  | Chi^2 | 0,000 | 0,159 | 0,000 | 0,000 | 0,000 | 0,000 |
|  | DF | 0 | 1 | 0 | 0 | 0 | 0 |
|  | Pr(chi^2 >) | 1,000 | 0,690 | 1,000 | 1,000 | 1,000 | 1,000 |
|  | Pr.exact | 1 | 1 | 1 | 1 | 1 | 1 |
| 11R | A | 2 | 3 | 1 | 1 | 2 | 1 |
|  | Ho | 0,07 | 0,07 | 0,00 | 0,00 | 0,03 | 0,00 |
|  | He | 0,06 | 0,07 | 0,00 | 0,00 | 0,03 | 0,00 |
|  | Chi^2 | 0,036 | 0,036 | 0,000 | 0,000 | 0,009 | 0,000 |
|  | DF | 1 | 3 | 0 | 0 | 1 | 0 |
|  | Pr(chi^2 >) | 0,850 | 0,998 | 1,000 | 1,000 | 0,926 | 1,000 |
|  | Pr.exact | 1 | 1 | 1 | 1 | 1 | 1 |
| 12R | A | 1 | 1 | 2 | 1 | 2 | 1 |
|  | Ho | 0,00 | 0,00 | 0,04 | 0,00 | 0,12 | 0,00 |
|  | He | 0,00 | 0,00 | 0,04 | 0,00 | 0,11 | 0,00 |
|  | Chi^2 | 0,000 | 0,000 | 0,010 | 0,000 | 0,097 | 0,000 |
|  | DF | 0 | 0 | 1 | 0 | 1 | 0 |
|  | Pr(chi^2 >) | 1,000 | 1,000 | 0,920 | 1,000 | 0,755 | 1,000 |
|  | Pr.exact | 1 | 1 | 1 | 1 | 1 | 1 |
| 13R | A | 1 | 1 | 1 | 1 | 1 | 1 |
|  | Ho | 0,00 | 0,00 | 0,00 | 0,00 | 0,00 | 0,00 |
|  | He | 0,00 | 0,00 | 0,00 | 0,00 | 0,00 | 0,00 |
|  | Chi^2 | 0,000 | 0,000 | 0,000 | 0,000 | 0,000 | 0,000 |
|  | DF | 0 | 0 | 0 | 0 | 0 | 0 |
|  | Pr(chi^2 >) | 1,000 | 1,000 | 1,000 | 1,000 | 1,000 | 1,000 |
|  | Pr.exact | 1 | 1 | 1 | 1 | 1 | 1 |
| 14R | A | 3 | 2 | 1 | 2 | 2 | 1 |
|  | Ho | 0,20 | 0,00 | 0,00 | 0,00 | 0,07 | 0,00 |
|  | He | 0,18 | 0,12 | 0,00 | 0,12 | 0,06 | 0,00 |
|  | Chi^2 | 0,185 | 15,000 | 0,000 | 15,000 | 0,018 | 0,000 |
|  | DF | 3 | 1 | 0 | 1 | 1 | 0 |
|  | Pr(chi^2 >) | 0,980 | 0,000 | 1,000 | 0,000 | 0,894 | 1,000 |
|  | Pr.exact | 1 | 0 | 1 | 0 | 1 | 1 |
| 15R | A | 2 | 3 | 4 | 3 | 5 | 2 |
|  | Ho | 0,59 | 0,52 | 0,17 | 0,48 | 0,41 | 0,52 |
|  | He | 0,44 | 0,56 | 0,30 | 0,49 | 0,72 | 0,50 |
|  | Chi^2 | 3,170 | 0,360 | 16,230 | 1,292 | 24,938 | 0,045 |
|  | DF | 1 | 3 | 6 | 3 | 10 | 1 |
|  | Pr(chi^2 >) | 0,075 | 0,948 | 0,013 | 0,731 | 0,005 | 0,832 |
|  | Pr.exact | 0 | 1 | 0 | 1 | 0 | 1 |
| 16R | A | 2 | 2 | 1 | 2 | 1 | 1 |
|  | Ho | 0,10 | 0,00 | 0,00 | 0,97 | 0,00 | 0,00 |
|  | He | 0,15 | 0,28 | 0,00 | 0,50 | 0,00 | 0,00 |
|  | Chi^2 | 3,580 | 30,000 | 0,000 | 26,254 | 0,000 | 0,000 |
|  | DF | 1 | 1 | 0 | 1 | 0 | 0 |
|  | Pr(chi^2 >) | 0,058 | 0,000 | 1,000 | 0,000 | 1,000 | 1,000 |
|  | Pr.exact | 0,25 | 0 | 1 | 0 | 1 | 1 |
| 17R | A | 2 | 2 | 3 | 2 | 4 | 3 |
|  | Ho | 0,36 | 0,04 | 0,18 | 0,43 | 0,14 | 0,50 |
|  | He | 0,46 | 0,04 | 0,25 | 0,46 | 0,31 | 0,59 |
|  | Chi^2 | 1,383 | 0,009 | 32,249 | 0,124 | 39,033 | 7,683 |
|  | DF | 1 | 1 | 3 | 1 | 6 | 3 |
|  | Pr(chi^2 >) | 0,240 | 0,923 | 0,000 | 0,724 | 0,000 | 0,053 |
|  | Pr.exact | 0,25 | 1 | 0 | 1 | 0 | 0,25 |
| 18R | A | 2 | 3 | 4 | 2 | 3 | 3 |
|  | Ho | 0,19 | 0,48 | 0,15 | 0,04 | 0,56 | 0,52 |
|  | He | 0,17 | 0,50 | 0,24 | 0,04 | 0,42 | 0,50 |
|  | Chi^2 | 0,281 | 0,825 | 54,012 | 0,010 | 3,994 | 0,729 |
|  | DF | 1 | 3 | 6 | 1 | 3 | 3 |
|  | Pr(chi^2 >) | 0,596 | 0,843 | 0,000 | 0,922 | 0,262 | 0,866 |
|  | Pr.exact | 1 | 1 | 0 | 1 | 0 | 1 |
| 19R | A | 2 | 2 | 2 | 1 | 1 | 2 |
|  | Ho | 1,00 | 0,67 | 0,00 | 0,00 | 0,00 | 0,63 |
|  | He | 0,50 | 0,44 | 0,18 | 0,00 | 0,00 | 0,43 |
|  | Chi^2 | 30,000 | 7,500 | 30,000 | 0,000 | 0,000 | 6,443 |
|  | DF | 1 | 1 | 1 | 0 | 0 | 1 |
|  | Pr(chi^2 >) | 0,000 | 0,006 | 0,000 | 1,000 | 1,000 | 0,011 |
|  | Pr.exact | 0 | 0 | 0 | 1 | 1 | 0 |
| 20R | A | 3 | 2 | 1 | 1 | 1 | 2 |
|  | Ho | 0,07 | 0,00 | 0,00 | 0,00 | 0,00 | 0,97 |
|  | He | 0,07 | 0,50 | 0,00 | 0,00 | 0,00 | 0,50 |
|  | Chi^2 | 0,037 | 29,000 | 0,000 | 0,000 | 0,000 | 25,262 |
|  | DF | 3 | 1 | 0 | 0 | 0 | 1 |
|  | Pr(chi^2 >) | 0,998 | 0,000 | 1,000 | 1,000 | 1,000 | 0,000 |
|  | Pr.exact | 1 | 0 | 1 | 1 | 1 | 0 |
| 21R | A | 3 | 1 | 2 | 2 | 3 | 2 |
|  | Ho | 0,37 | 0,00 | 0,37 | 0,23 | 0,70 | 0,37 |
|  | He | 0,42 | 0,00 | 0,30 | 0,21 | 0,48 | 0,30 |
|  | Chi^2 | 30,004 | 0,000 | 1,512 | 0,523 | 8,698 | 1,512 |
|  | DF | 3 | 0 | 1 | 1 | 3 | 1 |
|  | Pr(chi^2 >) | 0,000 | 1,000 | 0,219 | 0,469 | 0,034 | 0,219 |
|  | Pr.exact | 0 | 1 | 0,5 | 1 | 0 | 1 |
| 22R | A | 2 | 1 | 3 | 4 | 2 | 2 |
|  | Ho | 0,03 | 0,00 | 0,45 | 0,90 | 0,03 | 0,07 |
|  | He | 0,03 | 0,00 | 0,52 | 0,53 | 0,03 | 0,49 |
|  | Chi^2 | 0,009 | 0,000 | 30,378 | 76,861 | 0,009 | 21,341 |
|  | DF | 1 | 0 | 3 | 6 | 1 | 1 |
|  | Pr(chi^2 >) | 0,925 | 1,000 | 0,000 | 0,000 | 0,925 | 0,000 |
|  | Pr.exact | 1 | 1 | 0 | 0 | 1 | 0 |
| 23R | A | 1 | 1 | 1 | 1 | 2 | 1 |
|  | Ho | 0,00 | 0,00 | 0,00 | 0,00 | 0,03 | 0,00 |
|  | He | 0,00 | 0,00 | 0,00 | 0,00 | 0,03 | 0,00 |
|  | Chi^2 | 0,000 | 0,000 | 0,000 | 0,000 | 0,009 | 0,000 |
|  | DF | 0 | 0 | 0 | 0 | 1 | 0 |
|  | Pr(chi^2 >) | 1,000 | 1,000 | 1,000 | 1,000 | 0,926 | 1,000 |
|  | Pr.exact | 1 | 1 | 1 | 1 | 1 | 1 |
| 24R | A | 2 | 2 | 1 | 1 | 4 | 2 |
|  | Ho | 0,03 | 0,72 | 0,00 | 0,00 | 0,10 | 1,00 |
|  | He | 0,03 | 0,48 | 0,00 | 0,00 | 0,13 | 0,50 |
|  | Chi^2 | 0,009 | 7,633 | 0,000 | 0,000 | 28,503 | 29,000 |
|  | DF | 1 | 1 | 0 | 0 | 6 | 1 |
|  | Pr(chi^2 >) | 0,925 | 0,006 | 1,000 | 1,000 | 0,000 | 0,000 |
|  | Pr.exact | 1 | 0 | 1 | 1 | 0,25 | 0 |
| 25R | A | 2 | 3 | 1 | 2 | 3 | 2 |
|  | Ho | 0,11 | 0,86 | 0,00 | 0,14 | 0,96 | 1,00 |
|  | He | 0,10 | 0,53 | 0,00 | 0,13 | 0,55 | 0,50 |
|  | Chi^2 | 0,090 | 45,920 | 0,000 | 0,166 | 24,271 | 28,000 |
|  | DF | 1 | 3 | 0 | 1 | 3 | 1 |
|  | Pr(chi^2 >) | 0,765 | 0,000 | 1,000 | 0,684 | 0,000 | 0,000 |
|  | Pr.exact | 1 | 0 | 1 | 1 | 0 | 0 |
| 26R | A | 2 | 3 | 6 | 3 | 4 | 3 |
|  | Ho | 0,34 | 0,62 | 0,21 | 0,41 | 0,62 | 0,31 |
|  | He | 0,29 | 0,66 | 0,60 | 0,63 | 0,72 | 0,63 |
|  | Chi^2 | 1,259 | 7,322 | 64,939 | 11,933 | 6,542 | 19,251 |
|  | DF | 1 | 3 | 15 | 3 | 6 | 3 |
|  | Pr(chi^2 >) | 0,262 | 0,062 | 0,000 | 0,008 | 0,365 | 0,000 |
|  | Pr.exact | 0,25 | 0 | 0 | 0 | 0,25 | 0 |
| 27R | A | 2 | 3 | 2 | 3 | 5 | 3 |
|  | Ho | 0,50 | 0,35 | 0,00 | 0,42 | 0,65 | 0,12 |
|  | He | 0,41 | 0,61 | 0,07 | 0,59 | 0,71 | 0,24 |
|  | Chi^2 | 1,236 | 11,426 | 26,000 | 4,038 | 11,432 | 28,889 |
|  | DF | 1 | 3 | 1 | 3 | 10 | 3 |
|  | Pr(chi^2 >) | 0,266 | 0,010 | 0,000 | 0,257 | 0,325 | 0,000 |
|  | Pr.exact | 0,5 | 0 | 0 | 0,25 | 0,25 | 0 |
| 28R | A | 2 | 3 | 1 | 3 | 2 | 3 |
|  | Ho | 1,00 | 0,22 | 0,00 | 0,11 | 0,22 | 0,22 |
|  | He | 0,50 | 0,57 | 0,00 | 0,20 | 0,44 | 0,20 |
|  | Chi^2 | 9,000 | 10,960 | 0,000 | 18,000 | 2,250 | 0,141 |
|  | DF | 1 | 3 | 0 | 3 | 1 | 3 |
|  | Pr(chi^2 >) | 0,003 | 0,012 | 1,000 | 0,000 | 0,134 | 0,987 |
|  | Pr.exact | 0 | 0 | 1 | 0 | 0 | 1 |

Lab ID= population work IDs correspond with the Lab IDs in Table 1, A= counts of alleles, H_o_= observed heterozygosity, H_e_= expected heterozygosity, Chi^2= values of the Chi^2^ test, DF= degrees of freedom, Pr(chi^2>)= p-values of the Chi^2^ test, Pr.exact= p-values of the exact test. Locus names correspond with the primer set numbers.
