# Supplementary material for: Genetic diversity of Helosciadium repens (Jacq.) W.D.J. Koch (Apiaceae) in Germany, a Crop Wild Relative of celery
Source: Ecol Evol. 2019 Dec 17;10(2):875–90. doi: 10.1002/ece3.5947 (PMC6988547; doi:10.1002/ece3.5947)
Supplement: Supplementary file 3 [file ECE3-10-875-s003.docx]

Table S2: Percentage of individuals assigned to a DAPC cluster (*K* = 6) of 27 *Helosciadium repens* populations in Germany assessed with six microsatellites.

| Lab ID. | C1* | C2* | C3* | C4* | C5* | C6* |
| --- | --- | --- | --- | --- | --- | --- |
| 1R |  |  | 85 |  | 11 | 4 |
| 2R |  |  | 96 |  | 4 |  |
| 3R | 4 |  | 86 |  | 10 |  |
| 4R |  | 14 | 57 |  | 29 |  |
| 5R |  |  | 100 |  |  |  |
| 7R |  |  |  |  | 96 | 4 |
| 8R |  |  |  |  | 100 |  |
| 9R | 7 | 3 |  |  | 90 |  |
| 10R |  |  |  |  | 100 |  |
| 11R |  |  | 3 |  | 97 |  |
| 12R |  |  | 100 |  |  |  |
| 13R |  |  |  |  | 100 |  |
| 14R | 7 |  |  |  | 93 |  |
| 15R | 17 | 7 | 14 |  | 3 | 59 |
| 16R | 17 |  |  |  | 83 |  |
| 17R |  |  | 4 |  | 7 | 89 |
| 18R |  | 56 |  | 26 | 11 | 7 |
| 19R | 100 |  |  |  |  |  |
| 20R | 52 |  |  |  | 48 |  |
| 21R |  |  |  | 100 |  |  |
| 22R |  | 41 |  | 59 |  |  |
| 23R |  | 100 |  |  |  |  |
| 24R | 90 | 7 |  |  | 3 |  |
| 25R | 4 | 89 |  | 7 |  |  |
| 26R |  | 14 | 10 | 31 | 4 | 41 |
| 27R | 27 | 27 | 15 | 4 | 4 | 23 |
| 28R | 56 |  |  | 44 |  |  |

*Cluster assignments are proportional to the sample sizes (in %); Lab ID= population work IDs correspond with the Lab IDs in Table 1.
